# Ferric citrate and ferric EDTA but not ferrous sulfate drive amphiregulin-mediated activation of the MAP kinase ERK in gut epithelial cancer cells

**DOI:** 10.18632/oncotarget.24899

**Published:** 2018-03-30

**Authors:** Nathalie M. Scheers, Dora I.A. Pereira, Nuno Faria, Jonathan J. Powell

**Affiliations:** ^1^ Department of Biology and Biological Engineering, Chalmers University of Technology, Gothenburg, Sweden; ^2^ Elsie Widdowson Laboratory, Medical Research Council, Cambridge, UK; ^3^ Department of Pathology, University of Cambridge, Cambridge, UK; ^4^ MRC Unit The Gambia at the London School of Hygiene & Tropical Medicine, Fajara, Banjul, The Gambia; ^5^ Biomineral Research Group, Department of Veterinary Medicine, University of Cambridge, Cambridge, UK

**Keywords:** iron, ferric citrate, ferric EDTA, amphiregulin, pERK

## Abstract

Ferric chelates may be used as oral iron supplements or phosphate binders but both ferric citrate and ferric EDTA have been shown to promote tumor burden in murine models of colon cancer. Here we studied their effects on cancer cell growth, at typical supplemental iron levels encountered in the gastrointestinal tract (0.01-0.2 mM). Caco-2 and/or Hutu-80 cells were exposed to these forms of chelated iron or to ferrous sulfate and outcomes were assessed using cell proliferation assays, proteome profiler arrays, western blot, and ELISA. Ferric EDTA and ferric citrate increased cellular levels of the onco-protein amphiregulin and its receptor (EGFr) which in turn stimulated the activation of the MAP kinase ERK. Simultaneously, the expression of the negative Wnt regulator, DKK-1, increased suggesting that cell proliferation through the Wnt pathway may be less pronounced in the presence of ferric EDTA and ferric citrate, unlike for ferrous sulfate. Moreover, ferrous sulfate did not increase levels of cellular amphiregulin or EGFr. We conclude that *specific* iron compounds affect cell signaling *differently* and some may increase the risk of colon cancer advancement in an amphiregulin-dependent fashion. Further scrutiny of safe oral iron use is merited.

## INTRODUCTION

Different chemical forms of oral iron are widely used in the prevention and treatment of iron deficiency anemia [1] and some may also be used as ‘phosphate binders’ to control dietary phosphate absorption in patients with renal disease [2, 3]. However, certain of these, notably two different ferric iron chelates, ferric EDTA and ferric citrate, have been observed to promote colon cancer in mice [4–7]. In one study, oral administration of ferric EDTA drove ulcerative colitis-associated carcinogenesis in two murine models, namely DSS-induced colitis and interleukin-2 knockouts. These findings were in contrast to low-dose intraperitoneal injections of iron-dextran (6 or 12 mg/kg body weight) which did not significantly affect tumor incidence or number, indicating that it is not body iron status *per se* that exacerbates colon cancer growth. In another study, using the DSS model again, long-term oral administration of ferric EDTA, at the same level as background dietary iron (i.e. + 49 mg Fe/kg diet thereby doubling total Fe intake), markedly increased tumor incidence compared to controls or mice receiving i.v. iron [2]. The obvious conclusion for the differential effects of oral and parenteral iron is that oral iron temporarily accumulates in the colon, where transit times are relatively slow, and if it is bioavailable, then this may promote local cancer growth, oxidative stress, and DNA damage [8].

However, whether the carcinogenic effects observed *in vivo* are restricted to certain forms of chelated iron or, in fact, extend to soluble forms of iron *per se* is not clear. Indeed, iron uptake by colorectal cancers has been shown to be achieved through local upregulation of the iron transport protein, DMT1 and the ferric reductase DcytB [9], and so it could be the case that any iron that is a substrate for this transporter risks exacerbating colon cancer. At low concentrations, ferrous ions tend not to precipitate and are thus bioavailable, especially to DMT-1. Indeed, it is generally considered that iron from ferrous sulfate is more bioavailable than from ferric citrate or ferric EDTA although iron bioavailability is dependent upon the diet composition such that a diet rich in phytate might inhibit iron absorption from ferrous sulfate but less so from ferric EDTA [10] Indeed, based upon *in vitro* data, a case could be made for ferrous sulfate, as well as some forms of chelated iron, for aggravating large bowel cancer risk. In this respect, a common mutation in colorectal cancers involves the tumor suppressor adenomatous polyposis coli (APC) gene. Loss of the APC gene product leads to accumulation of nuclear β-catenin which activates Wnt target genes involved in promoting cell proliferation and tissue growth (reviewed in [11, 12]). Several cell models (e.g. Caco-2, Hutu-80, SW480) used in gut research have mutations affecting β-catenin/Wnt signaling pathways. Incubation studies lasting twenty-four hours with Caco-2 or SW480 cells have shown Wnt signaling to be up-regulated by 0.1 mM ferrous sulfate, as well as by the chelate hemin at 0.05 mM [5, 13, 14]. On the other hand, whilst, for the SW480 cell line, Xue et al report increases in expression of the STAT3 protein and phosphorylated STAT3, in response to 0.01 and 0.1 mM ferrous sulfate, Wnt signaling was not increased [15].

In summary, it is not understood whether all forms of ‘bioavailable’ iron exacerbate gut cancer cells, and if so, if the same mechanism is involved for different such iron forms. We studied these questions at the cellular level using ferric citrate, ferric EDTA and ferrous sulfate.

## RESULTS

### Amphiregulin is induced in response to ferric citrate and ferric EDTA

**Figure 1:**
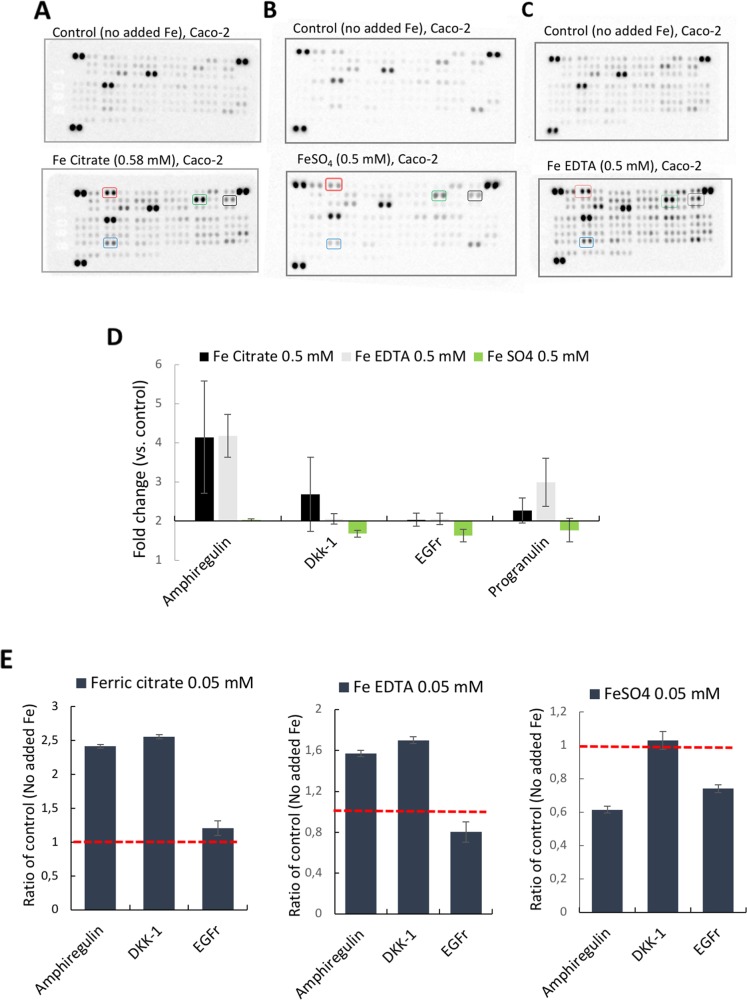
Proteome profiler™ arrays on human epithelial colorectal adenocarcinoma Caco-2 cells incubated with different iron compounds The cells were incubated with **(A)** Ferric citrate (0.5 mM), **(B)** Ferrous sulfate (0.5 mM), **(C)** Ferric EDTA (0.5 mM). Red box= Amphiregulin, Black box = EGFr, Green box= DKK-1, Blue box= Progranulin. **(D)** Quantative data based on the arrays (n=3). Fold changes ≥2 and signal ≥10% of the internal controls were considered as significant. **(E)** Bar graphs of array data for Amphiregulin (AREG), DKK-1, and EGFr at 0.05 mM, presented as ratio of control (Fold change) ± SD (n=2).

**Figure 2:**
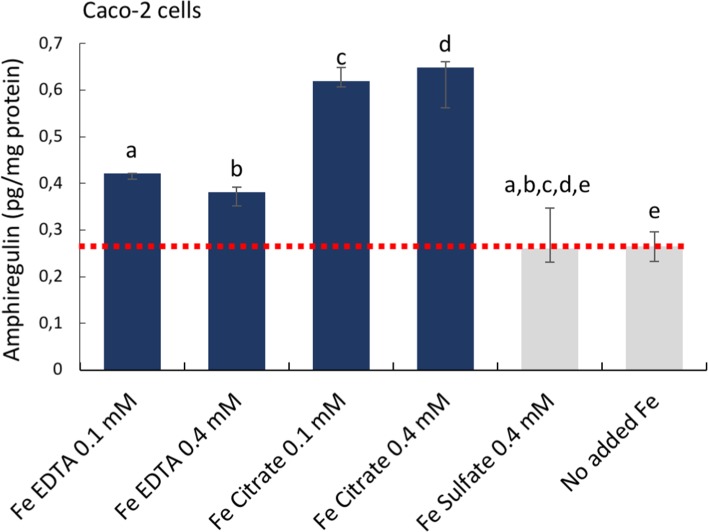
Cellular amphiregulin levels in human epithelial colorectal adenocarcinoma Caco-2 cells incubated with iron compounds Quantative data measured with Thermo Scientific™ hAREG ELISA kit. Data are presented as means, n=3 ± SD. The significance of the differences is expressed as letters a-e where a: p=2,5E^−7^, b: p=2,4E^−5^, c: p=1,14E^−10^, d: p=9.9E^−7^, e: p=0.6. Differences of p <0.05 were considered as significant.

First, targeted antibody arrays (proteome profiler™ arrays) were used to detect cancer-related protein levels on pooled triplicates of Caco-2 or Hutu-80 cells incubated with high concentrations (0.5-2 mM) of ferric citrate, which approximates to an equivalent 150-600 mg oral iron dose in a human subject (see Methods) as might be used for dietary phosphate binding in renal patients [3]. Notably, this induced cell-associated amphiregulin protein compared to controls (Figure 1A). Amphiregulin is a soluble paracrine growth factor and a ligand of the epidermal growth factor receptor (EGFr) (Supplementary Figure 1). Its major role is to promote proliferation and inhibit apoptosis during normal physiological developmental phases but abnormal induction has been implicated in epithelial cancers such as breast, ovary, lung, and colorectal cancers and is associated with activation of map kinases [16]. In addition, the levels of the amphiregulin target (EGFr) and the Wnt inhibitor DKK-1 were also increased in the presence of ferric citrate. Ferric EDTA at 0.5 mM also induced Amphiregulin, EGFr, and DKK-1 proteins whereas ferrous sulfate had no such effect (Figure 1B and 1C). Interestingly, Ferric citrate and ferric EDTA, but not ferrous sulfate, elevated protein levels of amphiregulin and DKK-1, even at low supplemental iron levels (0.05 mM) which would equate to an oral dose of 15 mg in humans (Figure 1E). However, at these low doses, the amphiregulin target, EGFr, was unaltered by all treatments (Figure 1E). In independent experiments, direct quantative data of cellular amphiregulin levels, in response to ferric citrate, ferric EDTA, and ferrous sulfate, supported the semi-quant proteome profiler data Figure 2.

### Elevated levels of amphiregulin are not related to cellular exposure to iron *per se*

One facile explanation for the results above is that the different forms of iron have different efficiencies of iron delivery to the cell and that this dictates the amphiregulin responses observed. We considered two separate angles. Firstly, the direct loading of cells by iron, in particular using the iron storage form, ferritin, as a read out for true intracellular iron targeting because measuring cell-iron content, grossly, does not distinguish cell-membrane-bound-iron from intracellular iron (i.e. it measures all cell-associated iron). Secondly, we considered how, at the molecular level, intracellular iron concentration might directly determine amphiregulin abundance.

**Figure 3:**
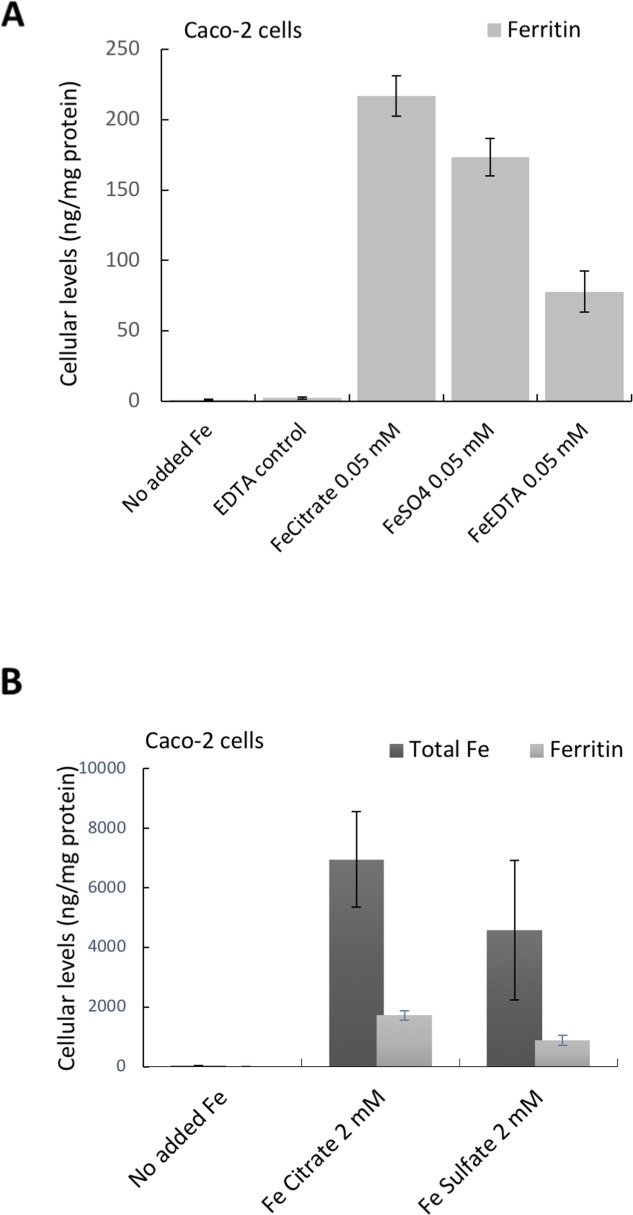
Cellular Ferritin **(A, B)** and total iron levels (B) in Caco-2 cells incubated with the different iron compounds. (A) Cellular ferritin levels of cells incubated with ferric EDTA (0.05 mM) was lower than in cells incubated with ferrous sulfate (0.05 mM). Data are presented as means ± SD (n=3). (B) Cellular levels of total iron and the corresponding ferritin levels in cells incubated with ferric citrate and ferrous sulfate (2 mM). Data are presented as means ± SD (n=4).

We compared amphiregulin levels at low iron exposure (0.05 mM) and the corresponding intracellular ferritin formation. In these cells, and as expected, amphiregulin was affected by the chelates used (iron in citrate or EDTA form) but not by ferrous sulfate. However, the increase in ferritin levels after iron exposure from the chelates did not follow the same trend as changes to amphiregulin (Figure 1E versus Figure 3A). Indeed, with low dose of iron supplementation the order of ferritin formation (iron bioavailability) was as follows: ferric citrate > ferric sulfate > ferric EDTA (Figure 3A), so this did not explain amphiregulin upregulation by the chelated iron forms only. To exclude the possibility that ferritin expression was induced by other signaling pathways, we compared the total iron content of cells incubated with high dose ferric citrate or ferrous sulfate (2 mM) with their ferritin levels. The ratio of iron:ferritin was similar for both iron forms, showing that ferritin levels were proportional to total iron content and very likely, therefore, to be directly related (Figure 3B).

**Figure 4:**
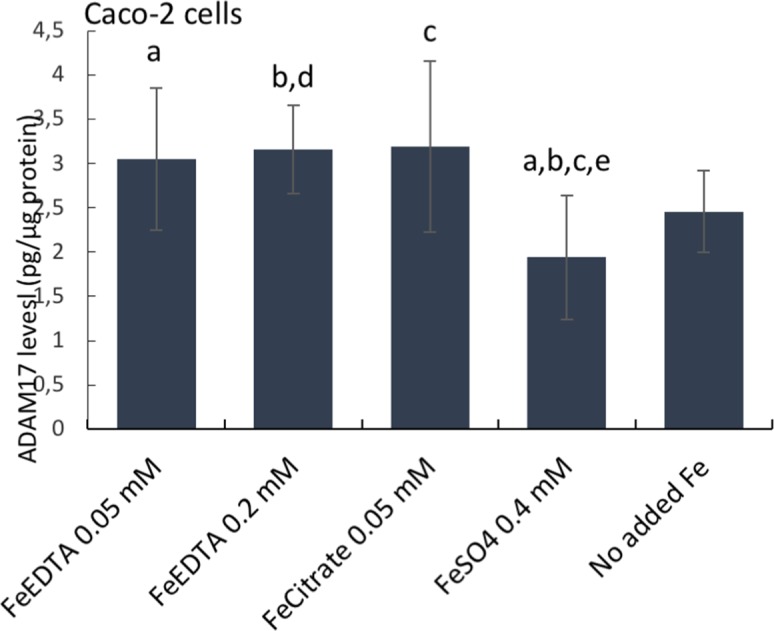
ADAM17 protein levels in human epithelial duodenum adenocarcinoma Caco-2 cells The cells were incubated with ferric citrate, ferric EDTA, ferrous sulfate, or control cells with no additional iron to the growth medium. ADAM17 levels were measured with the Thermo Scientific™ hTACE ELISA kit. Data are expressed as means ± SD (n=3). The significance of the differences is expressed as letters a-d where a: p=0.029, b: p=0.026, c: p=0.028, d: p=0.04, e: p=0.08. Differences of p <0.05 were considered as significant.

Next, we considered how cellular iron levels *per se* might influence amphiregulin at the molecular level. AREG mRNA does not contain iron responsive elements (IRE) and its translation is, therefore, not regulated by IRPs. Amphiregulin is a soluble protein produced by cleavage of an extracellular domain and, as such, it is regulated post-translationally. However, the cleavage of amphiregulin is mediated by different metalloproteinases such as ADAM17, which is regulated by O_2_ tension and HIF2, and the latter is related to iron influx in tumor cells [17]. However, cellular HIF2 levels were unchanged in response to the different iron supplements (data not shown), perhaps because IRPs have no IRE binding activity when intracellular iron levels are high and, in our assay, cells were not iron deficient. Notwithstanding, ADAM17 levels were unaffected in ferrous sulfate-treated cells (0.4 mM), versus no-added iron controls, but were significantly lower than for Ferric EDTA (0.05-0.2 mM) and ferric citrate (0.05 mM) treated cells (Figure 4). This supports the ideas that (a) only baseline levels of amphiregulin protein are expressed in cells incubated with ferrous sulfate (b) where iron-induced increases of cellular amphiregulin do occur, the relationship is with iron form, rather than iron levels *per se* and (c) there is a possible role for ADAM17 in the process, albeit in a HIF2-independent fashion.

### Cell proliferation

**Figure 5:**
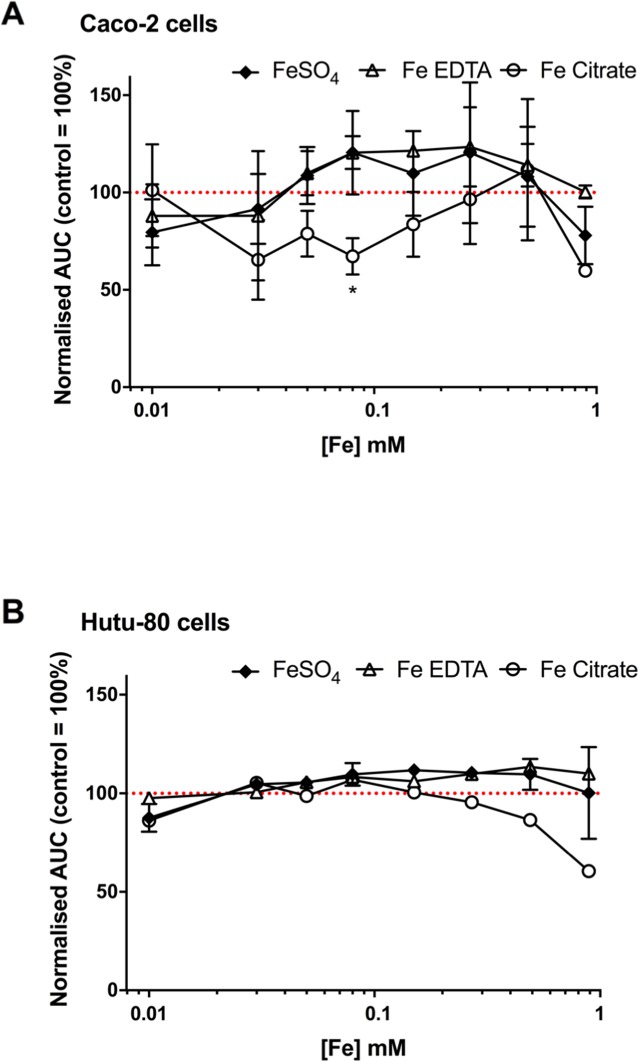
Confluence curves in the human epithelial colorectal adenocarcinoma cell line Caco-2 **(A)** and the human epithelial duodenum adenocarcinoma cell line Hutu-80 **(B)** with different iron compounds. Cells were treated for an average of 66 h with the indicated iron compounds in complete growth media containing 5% FBS. Data shown as area under the confluence curve for cells grown in media supplemented with each compound divided by the area under the curve for cells grown in un-supplemented media (i.e. media without any of the added iron compounds). Data are presented as mean with SD (*n* = 2 or 1 independent experiments with 3 replicates for each experiment).

We next considered whether the iron chelate-induced increase in amphiregulin was related to cell proliferation. In the first 72 hours post-seeding, different iron concentrations did not have a growth-promoting or growth-inhibitory effect on Caco-2 cells (Figure 5A) or Hutu-80 cells (Figure 5B). There was a suggestion for slight growth inhibitory effect of ferric citrate at higher concentrations, particularly in the Hutu-80 cells, and this could be an effect of the here observed higher bioavailability compared to ferrous sulfate and ferric EDTA (Figure 2) thereby ‘flooding’ the cell with potentially cytotoxic labile iron. However, this seemingly has no bearing on the cancer-promoting effect of ferric citrate, considering the results of prior murine studies [18] and, collectively, we conclude that amphiregulin regulation by chelated iron is unrelated to increased speed of cancer cell proliferation.

### Ferric citrate and ferric EDTA promote activation of ERK

**Figure 6:**
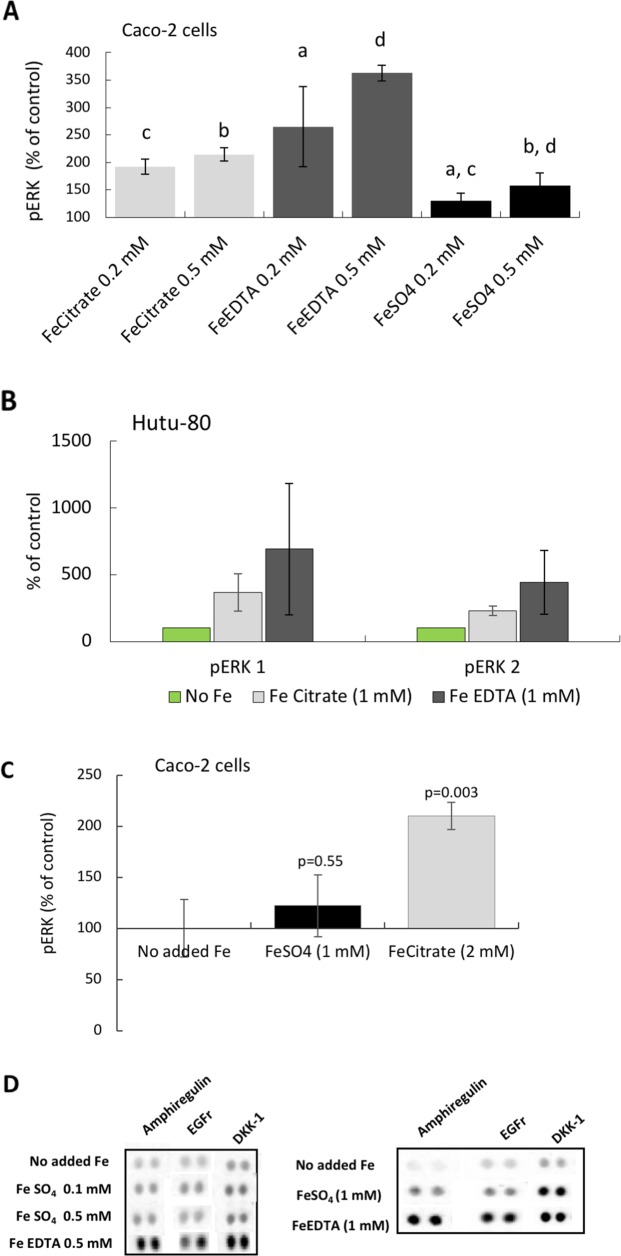
Phosphorylation of the MAP kinases ERK 1 and 2 in epithelial adenocarcinoma cells **(A)** Total phospho-ERK 1 and 2 levels in Caco-2 cells at iron levels that could be found in the human gut. Data are presented as Means ± SD (n=3) measured with instantOne™ phospho-ERK 1/2 assay. The significance of the differences is expressed as letters a-d where a: p=0.05, b: p=0.5, c: p=0.002, d: p=0.04. **(B)** Western blot data presented as a bar graph (n=2) ofphosphorylated ERK 1 and 2, respectively, in the human epithelia adenocarcinoma cell line Hutu-80 cells at slightly higher than normal gut concentration of iron (1 mM). **(C)** Total phospho-ERK 1 and 2 levels in Caco-2 cells at slightly higher iron concentration. **(D)** Protein Array data of amphiregulin, EGFr, and DKK-1 levels at comparative iron concentrations are presented for the purpose of comparison to the phosphorylation (activation) of ERK.

After establishing the induction of the EGFr substrate amphiregulin, we investigated the down-stream mechanism for activation of growth related pathways (see Supplementary Figure 1). Preliminary data suggest that the Wnt pathway might be down-regulated (by DKK-1; Figures 1A, 1C, 1D, 1E, 6D) and that the JAK/STAT pathway might be inactive (high BAD levels; data not shown), however, we found activity in the MAP kinase pathway. ELISA and Western blot data showed that The MAP kinases ERK 1 and 2 were activated (phosphorylated) in the presence of the iron chelates ferric citrate and ferric EDTA (0.2-0.5 mM in Caco-2 cells; Figure 6A, and in Hutu-80 cells (1 mM); Figure 6B). Thus, the increases in amphiregulin levels were associated with increased cellular levels of phosphorylated ERK (Figure 6A, 6B, 6C versus Figure 6D). Cells treated with high levels of ferrous sulfate (0.2-0.5 mM) did not respond with activation of ERK (Figure 6A) and even at supra-intestinal levels (1 mM) there was still only an insignificant trend to activate ERK (Figure 6C).

## DISCUSSION

Soluble forms of iron are widely used, both as nutritional supplements and also as therapeutics, the latter being mainly as oral iron replacement therapy or phosphate binders for patients with renal disease [2, 19]. The ingested dose for adults varies being, typically, up to 20 mg of iron for nutritional supplements, 30-120 mg iron for therapeutic supplements and as much as 630 mg iron for the purpose of phosphate binding [19]. The cellular work described here used soluble iron forms relevant to supplementation or phosphate binding, and iron doses that are anticipated to occur in the gut lumen following ingestion of this nutritional or therapeutic iron. The rationale behind our study is based on published data suggesting that soluble iron, particularly some forms of chelated ferric iron, increase tumorigenesis in the large bowel in murine studies [7, 9]. Here, we sought to investigate which mechanisms might be responsible for this observed effect on tumorigenesis, and if the effect is unique to the chelated iron forms used and thus varied depending on iron's chemical speciation. In this respect, there is no *in vivo* evidence for ferrous iron salts being a risk factor for enhanced tumorigenesis in the intestine, only studies in mouse models where diets were supplemented with either ferric citrate or ferric EDTA (see introduction). Indeed, we discovered that the iron chelates ferric EDTA and ferric citrate elevated the levels of the growth factor amphiregulin at all concentrations investigated (0.05-2 mM iron). This result was in contrast to ferrous sulfate which did not significantly increase amphiregulin levels at any iron level studied (0-1 mM). This was despite iron getting into cells, and being processed to upregulate ferritin, in all cases. In addition, we found that ERK phosphorylation was induced in the presence of the iron chelates only, indicating that the MAP kinase pathway was activated.

### Events preceding amphiregulin induction –does the form of iron matter?

The soluble active form of amphiregulin is produced by sequential proteolytic cleavage of its precursor ectodomain. There seem to be several mediators that can facilitate the cleavage and, among others, metalloproteinases (MMPs) have been suggested [20]. For example, the metalloproteinase ADAM17 has been associated with amphiregulin ectodomain cleavage [21]. In contrast to other EGFr ligands, amphiregulin is considered to be a low-affinity ligand to the EGF receptor [22]. Therefore, amphiregulin interaction with the receptor is less efficient than EGF or TGF alpha at negative feedback mechanisms such as downregulation and degradation of the receptor [23], so this is advantageous for cancer growth.

From the work reported here, amphiregulin induction appears to be a key event in the onset of the oncogenic MAP kinase pathway, driven by the ferric chelates investigated, but it is unlikely that iron *per se* induces the expression of amphiregulin, given the findings with ferrous sulfate. Our hypothesis as to why some forms of iron, such as these ferric chelates, may have a cancer promoting effect, is that ferric iron may be involved in the inducible cyclooxygenase (COX-2) pathway associated with inflammation. In colon cancer, PGE_2_ and EGF, products formed by the cyclooxygenase (COX-2) pathway, have been shown to induce amphiregulin production [24, 25]. There seem to be two possibilities for ferric iron to enter the cyclooxygenase pathway: either that ferric iron is involved directly in the oxidation of arachidonic acid and/or that ferric iron elevates the production of COX-2 and thereby promotes the conversion of arachidonic acid into prostaglandins (including PGE_2_) that might facilitate cancerous growth. In support of the idea that some forms of chelated ferric iron elevate COX-2 levels, or oxidize arachidonic acid directly, are studies showing that iron in the form of ferric nitrilotriacetate (FeNTA) increases the production of PGE_2_ (in rabbits) [26] and that oral administration of Deferiprone, which is a strong Fe (III) chelator, decreases levels of PGE_2_(in rats) [27]. The previous findings that ferric nitrilotriacetate-induced PGE_2,_ and our findings that ferric citrate and ferric EDTA induced amphiregulin, suggest that certain ferric chelates may be pro-cancerous. Simple ferric salts would not be available to enter the cell, as they precipitate forming solid phase oxo-hydroxides at intestinal/cellular pHs, whilst there is no evidence in the literature that ferrous sulfate would have an impact on COX-2 levels or PGE_2_ production which is supported by our study showing no effect on amphiregulin induction.

Radelescu *et al*. demonstrated that luminal iron (from ferric citrate added to the animal diets) strongly promoted tumorigenesis in a mouse model with a deletion of the tumor suppressor gene APC, which is a common mutation in colorectal cancers [14]. In addition, they investigated ferrous sulfate effects on a Wnt target protein, c-Myc [14], as well as, in another study with a Wnt reporter assay [13], on cell lines, with and without the APC gene. Overall, they found that ferrous sulfate amplified c-Myc protein levels in the non-functional APC cell lines. In our studies, ferrous sulfate did not induce the Wnt inhibitor DKK-1 in contrast to the two ferric chelates investigated, which supports the previous findings that cell proliferation may be mostly driven through the Wnt pathway in the presence of ferrous sulfate.

Finally, on this aspect of discussion, we note that whilst there is convincing evidence that certain ferric chelates are associated with pro-cancerous activity in animal models with nothing yet shown for ferrous iron salts *in vivo*, it is possible that this is simply explained by choice of murine diet compositions. Ferric citrate appears to be the iron fortificant mostly used (e.g. in diets TD80394 and AIN76A).

## MATERIALS AND METHODS

Ferrous sulfate heptahydrate (FeSO_4_) and ferric EDTA sodium salt solution (NaFeEDTA) were purchased from Sigma Aldrich. Ferrous sulfate was dissolved in acidified ultrapure water to produce a stock solution ([Fe]= 40 mM). Ferric EDTA solution (NaFeEDTA) was diluted in ultrapure water to produce a stock solution of 40 mM Fe. A stock solution of ferric citrate (FeCitrate) [Fe]= 8 mM, was produced by adding citric acid to ferric chloride on a 1:1 (Fe:Citrate) molar ratio. The solubility of iron in the ferric citrate stock solution was determined to be 88.3 +/− 2.4 % (n=3) by ultrafiltration and ICP analysis, but the iron solubility in growth medium may be higher since the iron concentration is lower. All stock solutions were filter sterilised (0.22 μm).

### Cell culture

Two different epithelial cancer cell lines were used: colorectal adenocarcinoma [Caco-2 (ATCC^®^ HTB37) and duodenum adenocarcinoma [Hutu 80 (ATCC^®^HTB40)]. The cells were grown in an incubator at 37°C, 5% CO_2_ and 95% air at a relative humidity of approximately 95%. The medium was changed every second day (except for weekends) and the cells were passaged at approximately 80% confluence. The cells were grown in MEM (E15-825; PAA, Pasching, Austria) with 10% FBS supplemented with Normocin™ (100 μg/ml; Invivogen) and before/during the experiments, the medium was supplemented with 5% FBS.

### Cell experiments

Caco-2 (p.31-40) and Hutu-80 cells (p. not retrievable) were seeded in 12-well plates (Corning, San Fransisco, CA, USA) at 150 000 or 200 000 cells/well. The medium (MEM 5% FBS) was supplemented with iron solutions (chelated iron was soluble), except for controls, at [Fe] between 0.05 mM-2 mM. The supplemented medium was aspirated after 48 hours of incubation and the cells were washed in PBS before lysis in RIPA buffer (Sigma Aldrich, Schnelldorf, Germany) containing Pierce phosphatase and protease inhibitors, EDTA-free (Thermoscinetific, Rockford, IL, US). Aliquots of cell lysates were analysed for ferritin (DRG, CA, US) and total protein (Pierce, Chicago, IL, USA)) according to the manufacturer's protocols. Total Fe was measured by inductively coupled plasma mass spectrometry using an Agilent 8800 Triple Quadrupole (ICP-MS/MS) coupled with an Integrated Sample Introduction System (ISIS-DS; Agilent Technologies, Cheshire UK). Calibration standards were matrix matched using pooled urine spiked with 50ppb to 5ppm iron. All samples, reference materials (Clinchchek and Seronorm) and calibration standards were diluted 20-fold in a solution containing 0.005% Triton™X-100, 0.05% TMAH (tetramethyl ammonium hydroxide) and 5 ppb Ge, which was used as internal standard. A mixture of hydrogen and oxygen was used as a reaction gas for the removal of analytical interferences and the main isotope of iron on mass (^56^Fe) was used for quantification.

### Proliferation assay

Working solutions of 2 mM Fe for each iron compound were prepared fresh on the day of the experiment by diluting the stock solutions of the different iron materials in complete cell growth medium (5% FBS). The 2mM working solutions were used in serial dilutions to achieve the 8 different iron concentrations for the proliferation assay (0.89, 0.49, 0.27, 0.15, 0.08, 0.05, 0.026, 0.015 mM [Fe]) in 96-well ImageLock cell culture plates. Control wells containing only complete growth medium without any extra iron compounds added were also prepared. The cells were seeded at a density of 10,000-20,000 cells/well and the plates were incubated in a Live Content Imaging Incubator (Incucyte ZOOM, Essen BioScience Ltd., UK). Images and confluence data were acquired every 3 hours for 66 hours (on average) post-seeding. Within each experiment, each iron concentration was tested in triplicate wells and the experiments were carried out on two separate occasions for Caco-2 cells (n=2) and on two or one occasion for the Hutu-80 cells (n=2 for FeSO_4_ and n=1 for Fe Citrate and Fe EDTA).

### Translation of *in vitro* [iron] into human dosing

The postprandial luminal concentration of ionisable iron after a standard meal containing 3.45 mg of iron has been reported as 10 μM [28], therefore, a supplemental single dose of 60-65 mg of Fe would correspond to almost 200 μM iron in the lumen whilst a dose of 600-650 mg Fe would correspond to almost 2 mM.

### Proteome profiling

Proteome profiler™ antibody arrays (Human XL Oncology Array Kit; R&D systems, MN; USA) for parallel determination of the relative levels of 84 human cancer-related proteins were used. Pooled triplicates of cell lysates were loaded (90 or 105 μg of total protein) to the arrays and the procedure was repeated for 2-4 experiments carried out on separate occasions. Development of the chemiluminescent signal was done using a ChemiDoc XRS+ (Bio-rad) and the membranes incubated with control and treatments to be compared, were processed and developed simultaneously to avoid differences in signal strength dependent on exposure times.

### ELISA

Phosphorylated ERK levels were measured with instantOne™ phospho-ERK 1/2 assay (Affymetrix, CA, USA) according to the instructions of the kit. 10 μl of cell lysates were loaded into each well and the measured A_450_ signal was normalized to protein content of each sample. Cellular ADAM17 and amphiregulin levels were measured with the Thermo Scientific™ Human Tace (ADAM17) Elisa kit and the Thermo Scientific™ Human Amphiregulin Elisa kit, respectively, according to the manufacturer's instructions. ADAM17 and amphiregulin levels were normalized to the cellular protein content of each sample.

### SDS PAGE and western blot

The cell lysates were diluted in Laemmli sample buffer with 2-mercaptoethanol and heated at 95 °C for 5 min. Samples (20 μg protein) were loaded on TGX-gels (Bio-rad) and run with Tris/glycine/SDS buffer at 200 V. After electrophoresis, the separated proteins were blotted to PVDF membranes using the Trans-Blot Turbo system with pre-packed transfer packs and the 3-min protocol (Bio-rad). After that, the blots were incubated in blocking buffer (Sigma Aldrich, Schnelldorf, Germany) at room temperature for 1 h. The primary antibody (r-α-human p-ERK; AF1018, R&D systems) was diluted in blocking buffer (0.2 μg/mL) and the membranes were incubated overnight at 4 °C. After washing, the blots were incubated with secondary antibody (0.5 μg/mL) and StrepTactin-HRP conjugate (1 μL/10 ml) for 1 h. After washing in PBS tween, the blots were added to a solution of luminol and peroxide buffer (Bio-rad) and the bands were detected by the ChemiDoc™ XRS+ system (Bio-rad) and analyzed with the software Image Lab™ 3.0.1 (Bio-rad).

### Statistics

Where applicable, data are presented as means ± SD (n=2-4). Ferritin, protein, ELISA, WB data were calculated using Microsoft^®^ excel for Mac version 15.36. For the proliferation assay data, the plots of confluence (%) vs time (h) were obtained for each iron compound and concentration using the Incucyte ZOOM software. Then, the area under each confluence curve (AUC) was determined, using GraphPad Prism 7.0, and plotted against the concentration for each test compound or control. Data is shown as AUC for each iron compound normalised against that of the control, plotted against iron concentration in Log_10_ scale (i.e. complete growth medium without supplemental iron added). Changes in the protein array data were considered significant if reaching a signal threshold of 10% of internal control and fold changes greater than 2.

## CONCLUSION

Ferric citrate and ferric EDTA induced amphiregulin production and the activation of the MAP kinase ERK while the Wnt inhibitor DKK-1 levels were high, but ferrous sulfate did not significantly increase amphiregulin or DKK-1 protein at supplemental/therapeutic levels of iron. These events suggest that different forms of iron may impact intestinal tumorigenesis differently. The chelates ferric citrate and ferric EDTA induce the oncogenic growth factor amphiregulin (a positively associated risk factor with colorectal cancer) and it should now be determined whether the ferrous sulfate impact on Wnt pathways *in vitro* translates to an oncogenic risk *in vivo*.

## SUPPLEMENTARY MATERIALS FIGURE



## Abbreviations

PGE2: Prostaglandin E2
IRE: Iron Response Element
IRP: Iron regulatory protein
ERK: Extracellular signal regulated kinase
MAP kinase: Mitogen activated protein
EDTA: Ethylenediaminetetraacetic acid
EGFr: Epidermal growth factor receptor
COX: Cyclooxygenase
i.v.: Intravenous
