# Supplementary material for: Ferric citrate and ferric EDTA but not ferrous sulfate drive amphiregulin-mediated activation of the MAP kinase ERK in gut epithelial cancer cells
Source: Oncotarget. 2018 Mar 30;9(24):17066–77. doi: 10.18632/oncotarget.24899 (PMC5908306; doi:10.18632/oncotarget.24899)
Supplement: Supplementary file 1 [file oncotarget-09-17066-s001.pdf]

Ferric citrate and ferric EDTA but not ferrous sulfate drive amphiregulin-mediated activation of the MAP kinase ERK in gut epithelial cancer cells

SUPPLEMENTARY MATERIALS

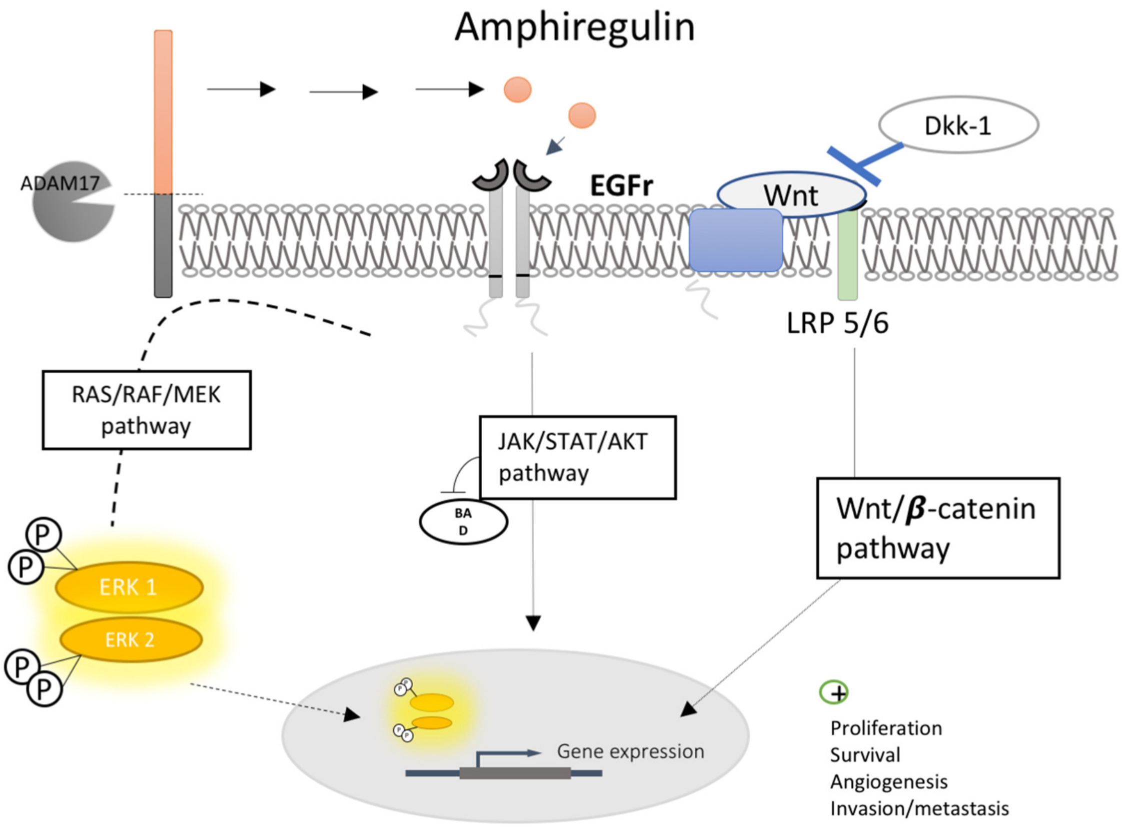

Supplementary Figure 1: Overview of amphiregulin activation of the MAP kinase pathway.
